# Flo5-1 and Nrg1 are involved in reversible pH-dependent flocculation in *Komagataella phaffii*

**DOI:** 10.1007/s00253-025-13562-7

**Published:** 2025-08-04

**Authors:** Sonakshi De, Gerhard Stadlmayr, Corinna Rebnegger, Diethard Mattanovich, Brigitte Gasser

**Affiliations:** 1 Austrian Centre of Industrial Biotechnology (acib), Vienna, Austria; 2 Department of Biotechnology and Food Science, Institute of Microbiology and Microbial Biotechnology, BOKU University, Vienna, Austria; 3 Christian Doppler Laboratory for Growth-Decoupled Protein Production in Yeast, Institute of Microbiology and Microbial Biotechnology, BOKU University, Vienna, Austria

**Keywords:** Yeast, Flocculation, Floc formation, pH, Sedimentation assay, Acidic pH, Flocculin genes, Transcriptional regulator

## Abstract

**Abstract:**

The non-conventional yeast *Komagataella phaffii* (syn *Pichia pastoris*) is a well-established host for biotechnological production processes, especially for recombinant protein production. Such processes are mostly run at neutral or slightly acid pH values between pH 5.0 and 6.5, but *K. phaffii* can grow also at lower or higher pH. Strikingly, we found that *K. phaffii* displays pH-dependent flocculation at pH 4, which is reversible when the cells are shifted to higher or lower pH. Six members of the flocculin (*FLO*) gene family were differentially regulated at pH 4.0. Of these, Flo5-1 was revealed to be crucially involved in the pH-triggered flocculation behavior, as cells lacking this flocculin (*flo5-1Δ*) settled at a much faster rate in the sedimentation assays. The transcriptional regulator Nrg1 was identified to negatively regulate this process, and cells overexpressing *NRG1* do not show the pH-dependent flocculation phenotype. In contrast to the model yeast *Saccharomyces cerevisiae*, neither the flocculin Flo11 nor the transcriptional activator Flo8 are involved in pH-dependent flocculation, once again highlighting the importance of studying transcriptional regulation mechanisms in non-conventional yeasts.

**Key points:**

• *Komagataella phaffii* *shows flocculation at pH 4, which is reversible at other pH*.

• *Six* *FLO* *genes are differentially expressed at low pH; flo5-1Δ flocculates stronger*.

*• K. phaffii* *Nrg1, but not Flo8, is involved in regulating pH-dependent flocculation*.

**Graphical Abstract:**

**Figure:**
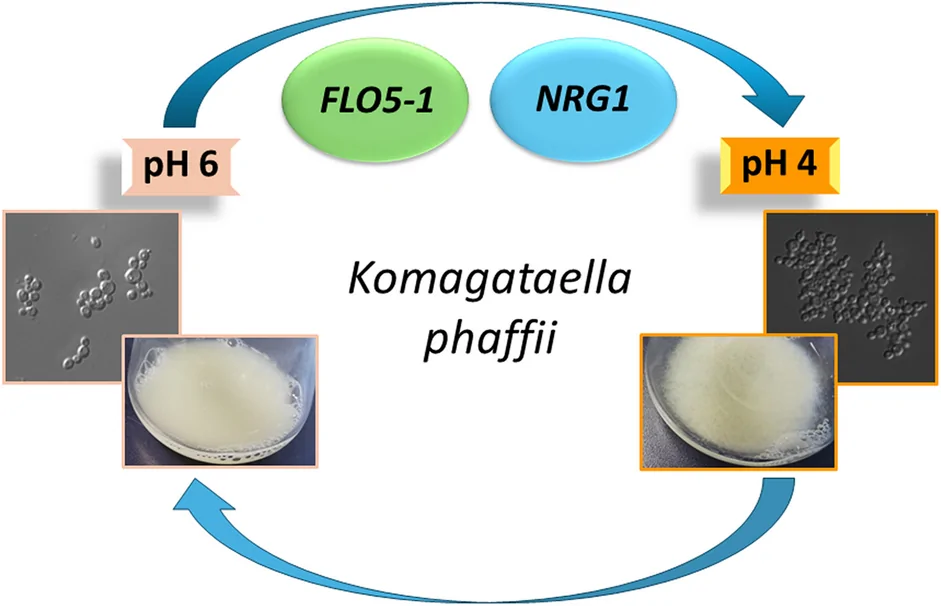

**Supplementary Information:**

The online version contains supplementary material available at 10.1007/s00253-025-13562-7.

## Introduction

Flocculation is a stress-response phenomenon displayed by yeasts upon being subjected to adverse environmental conditions, whereby yeast cells adhere to each other forming clumps or “flocs” that sediment out of the medium (Soares 2011). While the exact reason how flocculation can offer an advantage is still unclear, many theories for the same have been proposed. One such theory states that floc formation ensures that cells in the interior are protected from the harsh environmental conditions, with only the cells on the outer layer being sacrificed (Stratford and Assinder 1991; Smukalla et al. 2008). Another theory states that during nutrient-limiting conditions, autolysis of some cells in the flocs provides nutrition for adjacent cells (Stratford and Assinder 1991). Flocculation has also been shown to provide suitable conditions for mating within the flocs (Goossens et al. 2015). Apart from being an important stress-response mechanism, flocculation has provided an interesting model to study possible cooperative behavior in yeasts.

It has been reported that families of adhesin proteins are at the core of flocculation and other adhesion-related phenotypes. Generally, adhesins share a common structure with an N-terminal flocculin domain followed by a serine- and threonine-rich central domain containing tandem repeats and a C-terminal glycosylphosphatidylinositol (GPI) anchor (Willaert 2018). In baker’s yeast, the flocculation trait has since long been used in the brewing and ethanol production industry as a cost-effective method for biomass separation (Soares 2011). Considering the advantage that flocculating *Saccharomyces cerevisiae* strains provide in their wine/beer making applications, it is not surprising that the flocculation mechanism has been most widely studied and best characterized in this yeast. The *S. cerevisiae FLO* family consists of a group of subtelomeric genes, namely, *FLO1*, *FLO5*, *FLO9*, and *FLO10* and the non-subtelomeric *FLO11*. Additionally, the transcription factor Flo8 is a master regulator controlling the expression of most *FLO* genes. While Flo1, Flo5, Flo9, and Flo10 promote cell–cell adhesion and floc formation, Flo11 is mainly involved in pseudohyphal growth, invasive growth, and biofilm formation. Flo1, Flo5, Flo9, and Flo10 contain an N-terminal PA14 lectin domain which is responsible for cell–cell interaction (Teunissen and Steensma 1995; Caro et al. 1997; Guo et al. 2000; Lambrechts et al. 1996; Lo and Dranginis 1998; Van Mulders et al. 2009).

One of the environmental factors that can trigger flocculation in yeast is changes in pH, although the effect of pH is quite ambiguous. In *S. cerevisiae*, it has been shown that flocculation can occur over a wide range of pH, depending on the strain. While slightly acidic pH, in the range of 3.5–5.8, is optimal for yeast flocculation, some strains are known to flocculate better at higher pH (Soares et al. 1994; Jin et al. 2018). Extreme conditions of pH result in the dispersion of flocs, which could be due to structural alteration of the flocculins (Jin et al. 2018; Jin and Speers 2018).

The methylotrophic yeast and popular protein production host *Komagataella phaffii* (syn *Pichia pastoris*) also exhibits pH-dependent flocculation and tends to display flocculation at pH values of around 4.0. The mechanisms underlying this phenomenon are so far unstudied in *K. phaffii*. In a previous study, we have shown that the *FLO* gene family of *K. phaffii* consists of 13 gene members and that several of them (*FLO11*, *FLO400*, *FLO5-1*) are responsible for the onset of pseudohyphal growth in slow growth conditions in glucose-limited chemostats (De et al. 2020). Furthermore, three Flo proteins from the related yeast *Komagataella pastoris* have been structurally characterized (Kock et al. 2018; Brückner et al. 2020). In contrast, their involvement in the flocculation phenotype has not yet been explored. Therefore, this study aims at gaining a better understanding of low pH-triggered flocculation in *K. phaffii* and investigates the role(s) that members of the *FLO* gene family play in this phenotype.

## Materials and methods

### Strains and strain creation

All *K. phaffii* strains used in this study are based on the wild-type strain CBS7435 and listed in Supplementary Table S1. The CBS7435 *flo8Δ* used here was the same strain used in De et al. (2020), which is the strain described in Rebnegger et al. (2016) from which the antibiotic resistance cassette was removed.

The deletion strains *flo11Δ* and *flo5-1Δ* were created in our previous study (De et al. 2020). The deletion strains *flo5-2Δ*, *flo400Δ*, and *nrg1Δ* were also created using the split-marker cassette method (Fairhead et al. 1996; Gasser et al. 2013), whereby the target genes were replaced by an expression cassette encoding an antibiotic selection marker. Flanks of around 1000 bp of homologous regions, upstream and downstream of the target gene, were used for integration. The expression cassette for the selection marker, along with the flanking homologous regions, was assembled into a vector using Golden Gate Assembly (Prielhofer et al. 2017). Finally, this vector was used as a PCR template to generate two fragments with an overlap in the marker gene sequence. Five hundred nanograms of these two fragments was transformed into electrocompetent *K. phaffii* strains using the established protocol (Gasser et al. 2013).

All PCR amplifications for generation of the cassettes were carried out using Q5® High Fidelity DNA polymerase (New England Biolabs, Frankfurt am Main, Germany). Deletions were confirmed by colony PCR using forward and backward primers located outside the flanking homologous regions, using OneTaq DNA polymerase (New England Biolabs, Frankfurt am Main, Germany). All primer sequences are given in the Supplementary Tables S2 and S3.

For the* NRG1*-OE strain, the *NRG1* gene was expressed constitutively under the control of P_GAP_ and the terminator RPS25Att. The entire expression cassette was cloned into a plasmid by Golden Gate cloning for integration into the *K. phaffii* genome. The *NRG1* gene, flanked with *Bsa*I restriction sites and fusion sites BC, was amplified from the CBS7435 genomic DNA using Q5® DNA polymerase. The P_GAP_ (flanked with *Bsa*I restriction sites and fusion sites AB) and the transcription terminator *RPS25A*tt (flanked with *Bsa*I restriction sites and fusion sites CD) were amplified directly from available plasmids containing these sequences. The three fragments were assembled together in the BB3rN_AD (nourseothricin resistance) plasmid. For the *FLO5-1*-OE strain, the *FLO5-1* gene was expressed under the control of P_*PFK300*_ and terminator *RPS3*tt. The *FLO5-1* gene, flanked with *Bsa*I restriction sites, was amplified from the CBS7435 genomic DNA using Q5® DNA polymerase. The backbone containing the promoter, the terminator, the *RGI1* locus integration site, and the nourseothricin resistance cassette were amplified using Q5® DNA polymerase and assembled via Golden Gate cloning from an existing plasmid (Prielhofer et al. 2017). The assembled plasmids were linearized with *Asc*I in the *RGI1* locus for genomic integration and transformed into competent *K. phaffii* CBS7435. Correct integration was verified using colony PCR using primers as given in the Supplementary Table S3.

### Cultivation in acidic pH

For cultivation at acidic pH (3.0 and 4.0), minimal ASM medium was prepared containing (per L) 6.3 g (NH_4_)_2_HPO_4_, 0.8 g (NH_4_)_2_SO_4_, 0.49 g MgSO_4_* 7H_2_O, 2.64 g KCl, 0.0535 g CaCl_2_*2H_2_O, 22 g citric acid monohydrate, 1470 µL trace element solution, and 4 mL biotin stock solution (0.1 g L^−1^), supplemented with 2% glucose as a carbon source. The trace element solution contained per liter 5.0 g FeSO_4_·7H_2_O, 20.0 g ZnCl_2_, 6.0 g CuSO_4_·5H_2_O, 3.36 g MnSO_4_·H_2_O, 0.82 g CoCl_2_*6H_2_O, 0.2 g Na_2_MoO_4_·2H_2_O, 0.08 g NaI, 0.02 g H_3_BO_3_, and 5.0 mL H_2_SO_4_ (95–98% w/w). The media was aliquoted in three equal parts, and the pH was set to 6.4, 4.0, and 3.0. Depending on the required pH, NH_4_OH and/or KOH was used.

For the cultivation, an overnight preculture was prepared in YPD containing the respective antibiotic (either 500 µg/mL geneticin or 100 µg/mL nourseothricin). The next day, the OD_600_ of the preculture was measured, and 10 mL minimal medium was inoculated with the preculture so that the final OD_600_ of the main culture was about 4.0. The main culture was incubated overnight at 25 °C with shaking at 180 rpm. The next day, the images from the main culture flasks were taken to document the level of flocculation. Additionally, cells were also checked under the microscope. The main culture was also used for sedimentation assays.

For fixing of cells for transcriptomics studies, samples were added in a 2:1 ratio to precooled fixing solution containing 5% (vol/vol) phenol in absolute ethanol. These were then centrifuged for 1 min at 10,000 × g and 4 °C; afterwards, the supernatant was removed, and the pellet was stored at − 80 °C until further processing.

### Sedimentation assay

The sedimentation assay performed for quantification of settling was adapted from Stratford and Assinder (1991). Overnight cultivation of the main culture was carried out at the desired pH as described in the previous section with the exception of cultures intended for investigating metal ion dependence as well as the effect of *FLO5-1* overexpression, which were inoculated at an OD_600_ of about 1 and resuspended in fresh media approximately 2 h before samples were harvested for the sedimentation assay. Right before the sedimentation assay, the OD_600_ of the main culture was measured and cells corresponding to 25 OD_600_ units were harvested. These cells were resuspended in 5 mL minimal ASM media of the same pH as the main culture, contained in sealed glass test tubes. The test tubes were vortexed vigorously for about 10 s to resuspend the cells, and then, they were allowed to stand undisturbed. At specific times, samples were drawn from just below the meniscus, and the OD_600_ was measured and documented. The pH of the medium was checked at the start and end of the assay. Flocculation was quantified for each strain in four independent sedimentation assay experiments. For every sedimentation assay carried out, the wild-type was always included as a control. For assessing metal ion dependence, cells were resuspended in 0.1 M citrate buffer (CB) containing either 5 mM ethylenediaminetetraacetic acid (EDTA) or 5 mM CaCl_2_.

### RNA extraction and qRT-PCR

The ethanol/phenol fixed cells were thawed and centrifuged, and the fixing solution was removed. The cells were then resuspended in 1 mL TRI reagent (Sigma-Aldrich, Darmstadt, Germany) and transferred to 2-mL screwcap tubes containing 500 µL of acid-washed 500-µm glass beads. They were then lysed in a FastPrep®−24 equipment (MP Biomedicals, Irvine, CA, USA) for 5 × 40 s cycles, with a 2 min rest on ice in between. RNA extraction and qRT-PCR were carried out as described in De et al. (2020) using the primers given in Supplementary Table S4.

## Results

### pH-dependent flocculation of *K. phaffii* peaks at pH 4.0 and is reversible

**Fig. 1:**
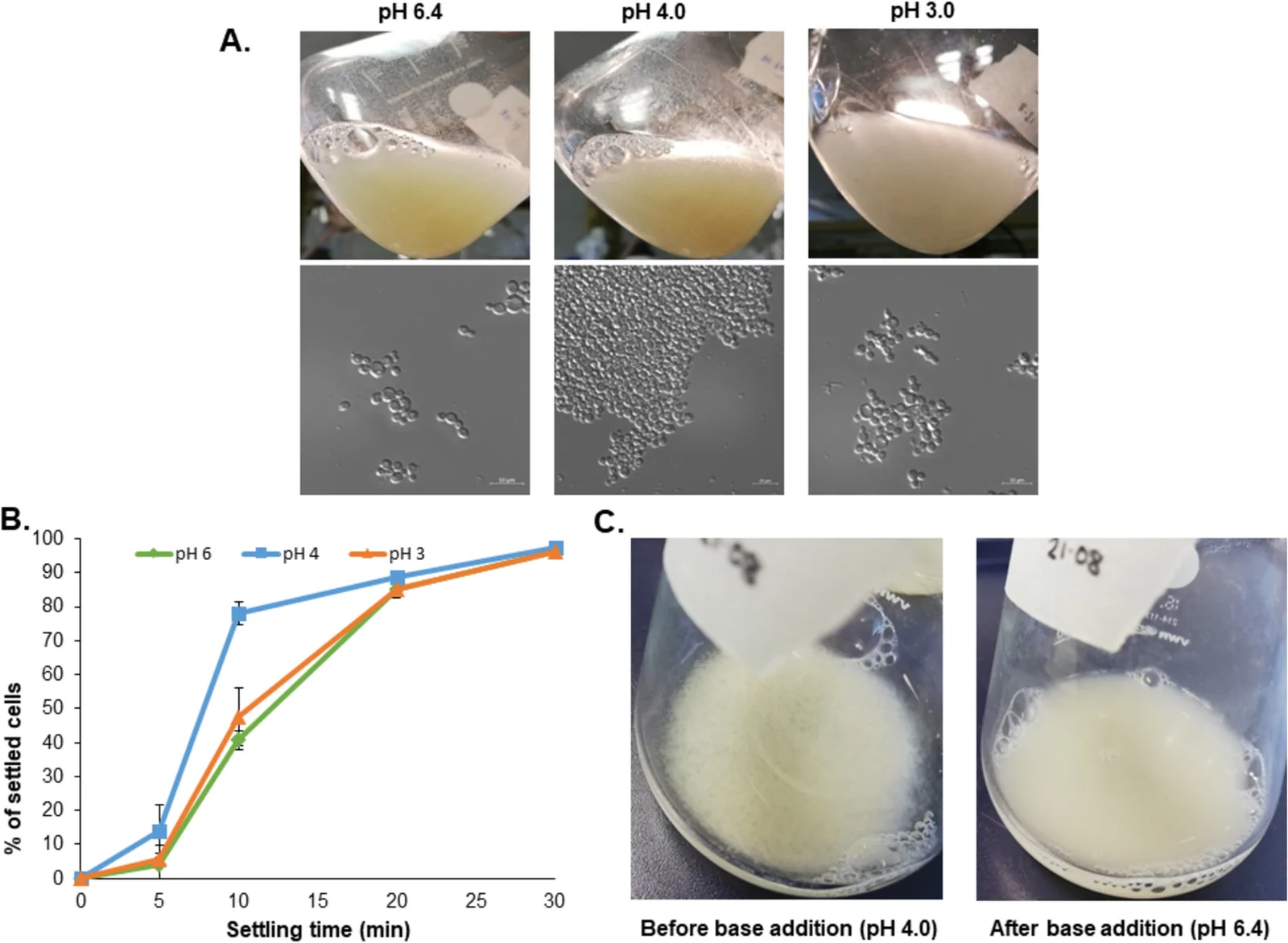
pH-dependent flocculation in *K. phaffii* wild-type CBS7435. **A** Flocculation observed after overnight cultivation in minimal medium set to pH 6.4, pH 4.0, or pH 3.0. The top panel shows the flocculation as observed after cultivation in a shake flask, and the bottom panel shows microscopic images from samples taken from the same flasks. **B** Sedimentation assay comparing the rate of sedimentation of wild-type *K. phaffii* CBS7435 at different pH. Error bars show standard deviation of four individual experiments. **C** pH-dependent flocculation can be reversed by raising the pH of the medium

To investigate the effect of low pH on *K. phaffii*, the wild-type strain CBS7435 was cultivated in minimal medium that was set to three different pH values: pH 6.4, pH 4.0, and pH 3.0. For standard cultivations of *K. phaffii*, it is common to use a pH of around 6.0–6.5, so pH 6.4 served as a control in our experiments. After overnight cultivation at the different pH values, the degree of flocculation was checked through microscopic images and sedimentation assay. As can be seen in Fig. 1A, there seems to be only some amount of cell aggregation at pH 6.4 and 3.0, while the maximum flocculation is observed at pH 4.0. The sedimentation assay (Fig. 1B) confirms this observation since the fastest rate for settling of the cells is observed at a pH of 4.0. It was also observed that raising the pH of the medium to 6.4 again is sufficient to remove the flocs, indicating that such a kind of flocculation is reversible (Fig. 1C).

Consequently, further cultivations for studying the flocculation phenotype were carried out at pH 4.0.

**Fig. 2:**
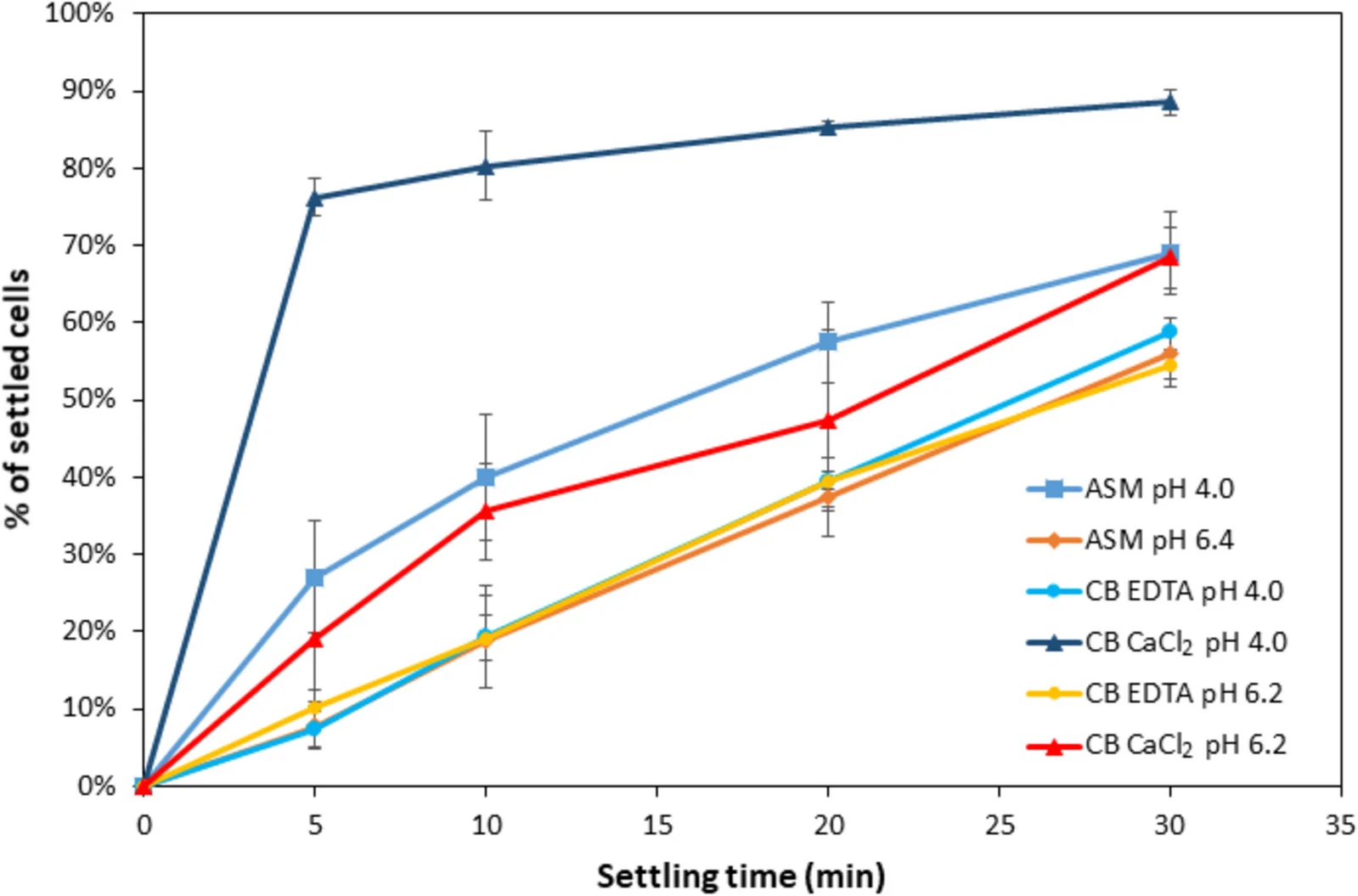
Impact of Ca^2+^ ions on pH-dependent flocculation in *K. phaffii *wild-type CBS7435. Sedimentation assay comparing the rate of sedimentation of wild-type *K. phaffii* CBS7435 at different pH in the presence of 0.36 mM Ca^2+^ (ASM minimal medium), or 5 mM Ca^2+^ (CB CaCl_2_) or 5 mM EDTA (CB EDTA) in 0.1 M citrate buffer (CB). Error bars show standard deviation of three individual experiments

Next, we analyzed if flocculation was dependent on the presence of metal ions. Therefore, the cells were resuspended in citrate buffer containing either 5 mM EDTA or 5 mM Ca^2+^ prior to the sedimentation assay. While the addition of the chelating agent EDTA had no impact at pH 6.4 and a moderate effect at pH 4.0, flocculation was strongly accelerated when further Ca^2+^ ions were added. This effect was more evident at pH 4.0 than at pH 6.4 (Fig. 2).

### Six *FLO* genes are differentially regulated upon cultivation at pH 4.0

**Fig. 3:**
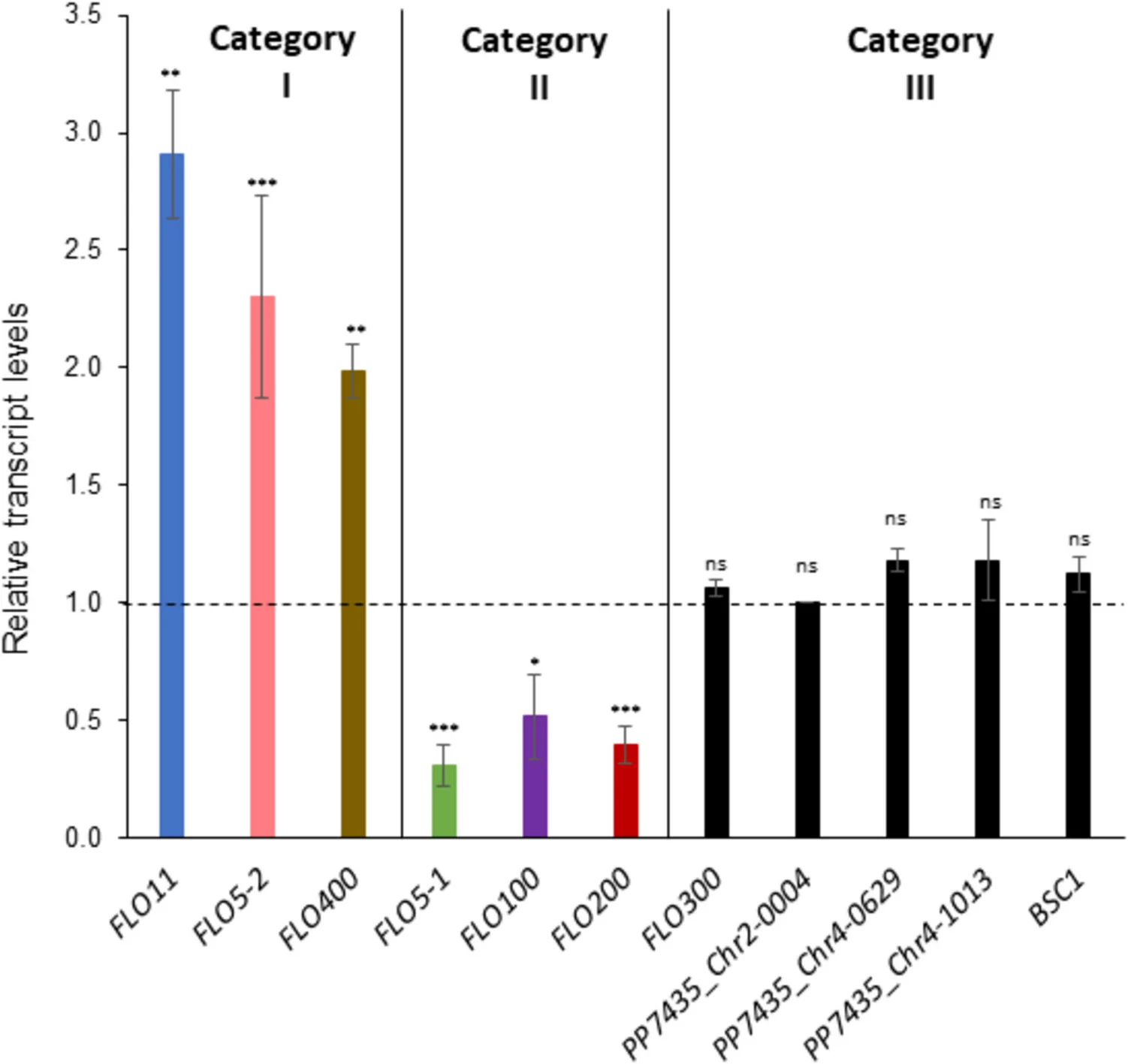
Relative transcript levels of *K. phaffii FLO *gene family members at pH 4.0 compared to pH 6.4. qRT-PCR data showing the level of expression of the three different categories of *FLO* genes (categories I, II, and III) in the wild-type strain after overnight cultivation at the required pH. Cultivations were carried out in two biological replicates. All expression levels were normalized to the expression of the housekeeping gene *ACT1* and then compared to the expression of the gene in the wild-type strain cultivated at pH 6.4, which was set to 1 (represented by the dashed line). Asterisks above the bars denote statistical significance of gene expression levels in comparison with the reference sample (*p*-values calculated by Student’s *t*-test; ns (not significant): *p*-value > 0.05, *p-value < 0.05, **p-value < 0.01, and ***p-value < 0.001)

In order to investigate the involvement of *FLO* genes in conferring the flocculation phenotype in *K. phaffii*, we looked for differential regulation of these genes when *K. phaffii* CBS7435 wild-type was cultivated at pH 4.0 compared to cultivation at pH 6.4, by quantitative real-time PCR (qRT-PCR). The gene identifiers and domain architecture of the proteins encoded by the 11 investigated *FLO* genes are found in Supplementary Fig. S1. As we were most interested in the flocculation behavior occurring during *K. phaffii* cultivations, all further assays were carried out in standard minimal media containing 0.36 mM Ca^2+^. Based on their regulation patterns (Fig. 3), the *FLO* genes can be grouped into three categories: The genes in category I comprising *FLO11*, *FLO5-2*, and *FLO400* are higher expressed at pH 4.0 compared to pH 6.4. The genes in category II comprising *FLO5-1*, *FLO100*, and *FLO200* are expressed lower at pH 4.0 compared to pH 6.4. Finally, the remaining genes (*FLO300*, *PP7435_Chr4-0629*, *PP7435_Chr4-1013*, *PP7435_Chr2-0004*, and *BSC1*) belong to category III, which show no difference in expression between the two conditions and were omitted from further investigations.

### In *K. phaffii*, the transcription factor Nrg1 negatively regulates pH-dependent flocculation while Flo8 does not seem to play a role in flocculation

Next, we wanted to investigate if the transcription factor Flo8 is involved in the low pH-triggered flocculation phenotype in *K. phaffii*. Flo8 has been described as a master activator of many *FLO* genes in *S. cerevisiae* (Liu et al. 1996). The Flo8 homolog in *K. phaffii* has previously been implicated with regulation of several *FLO* genes during pseudohyphae formation, and its absence prevented the elongation phenotype (Rebnegger et al. 2016; De et al. 2020). In *S. cerevisiae*, the absence of Flo8 prevents flocculation (Kobayashi et al. 1996; Fichtner et al. 2007).

**Fig. 4:**
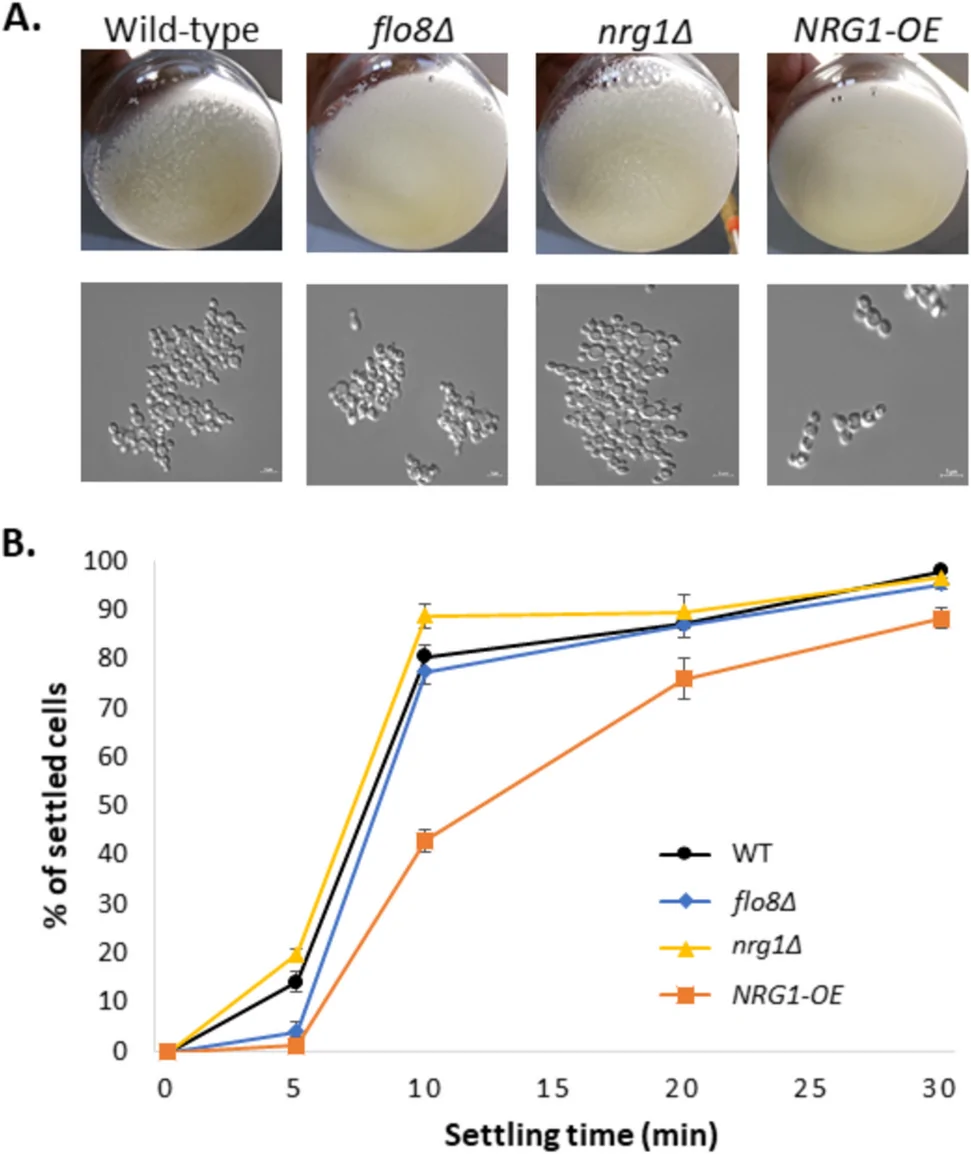
Flocculation in *K. phaffii* wild-type, *flo8Δ*, *nrg1Δ*, and* NRG1*-OE cells after overnight cultivation at pH 4.0. **A** The top panel shows the flocculation as observed after cultivation in shake flasks, and the bottom panel shows microscopic images from samples taken from the same flasks. **B** Sedimentation assay comparing the rate of sedimentation of *K. phaffii* wild-type (WT), *flo8*Δ, *NRG1*-OE, and *nrg1*Δ cells at pH 4.0

We cultivated *K. phaffii flo8*Δ and CBS7435 wild-type at pH 4.0 and pH 6.4 and took pictures of the flasks as well as microscopic images of each strain (Fig. 4A). Additionally, the sedimentation test was performed at pH 4.0 as described in the “Materials and methods” section (Fig. 4B).

In contrast to *S. cerevisiae*, *K. phaffii* Flo8 does not seem to be involved in flocculation, since the *flo8*Δ strain showed floc formation and a sedimentation behavior similar to the wild-type at pH 4.0 (Fig. 4). As can be seen in Fig. 4B, there is only a slight difference in sedimentation rate at the second time point between the wild-type and the *flo8*Δ strain. So, we concluded that Flo8 does not have a significant impact on pH-triggered flocculation in *K. phaffii*. This also corroborates our previous observation in glucose-limited chemostats where the *K. phaffii flo8*Δ strain showed a similar degree of flocculation as the wild-type, while pseudohyphae formation was completely prevented (De et al. 2020).

Nrg1 is a Cys_2_His_2_-type zinc finger transcriptional repressor shown to be involved in glucose- and glycerol-mediated repression of methanol utilization genes in *K. phaffii* (Wang et al. 2016). In *S. cerevisiae*, along with Nrg2, Nrg1 is also known to mediate other stress signals including filamentous growth, nitrogen depletion, heat and cold shock, osmotic stress, and acidic and alkaline pH (Vyas et al. 2005). A previous publication from our research group had identified *K. phaffii* Nrg1 (PAS_chr3_1242) as a factor enhancing recombinant protein secretion when overexpressed (Stadlmayr et al. 2010). We also observed that the *K. phaffii* X-33 strains overexpressing *NRG1* displayed reduced flocculation when cultivated at an acidic pH.

Considering the information that Nrg1 has been implicated in the regulation of *FLO11* in *S. cerevisiae* (Kuchin et al. 2002) and our previous observation that Nrg1 seemed to prevent low pH-triggered flocculation in *K. phaffii*, we decided to investigate the impact of this transcription factor further in the context of flocculation. For this purpose, we created *K. phaffii* strains with altered expression of Nrg1, namely *NRG1*-OE (overexpressing *NRG1* under control of the constitutive promoter P_*GAP*_) and *nrg1*Δ in CBS7435, and cultivated them at pH 4.0 and pH 6.4. Again, pictures showing the level of flocculation in each strain and microscopic images of the same were taken. Additionally, sedimentation assays at pH 4.0 were performed for *NRG1*-OE, *nrg1*Δ, and the wild-type strains simultaneously (Fig. 4).

As can be seen in Fig. 4B, the rate of sedimentation is significantly lower in the *NRG1*-OE than in the wild-type strain, corresponding to the reduced level of flocculation that was observed (Fig. 4A). *nrg1Δ* shows no difference in flocculation and sediments at a similar rate compared to the wild-type strain.

### qRT-PCR data identifies possible involvement of *FLO11*, *FLO5-1*, and *FLO5-2* in pH-triggered flocculation

**Fig. 5:**
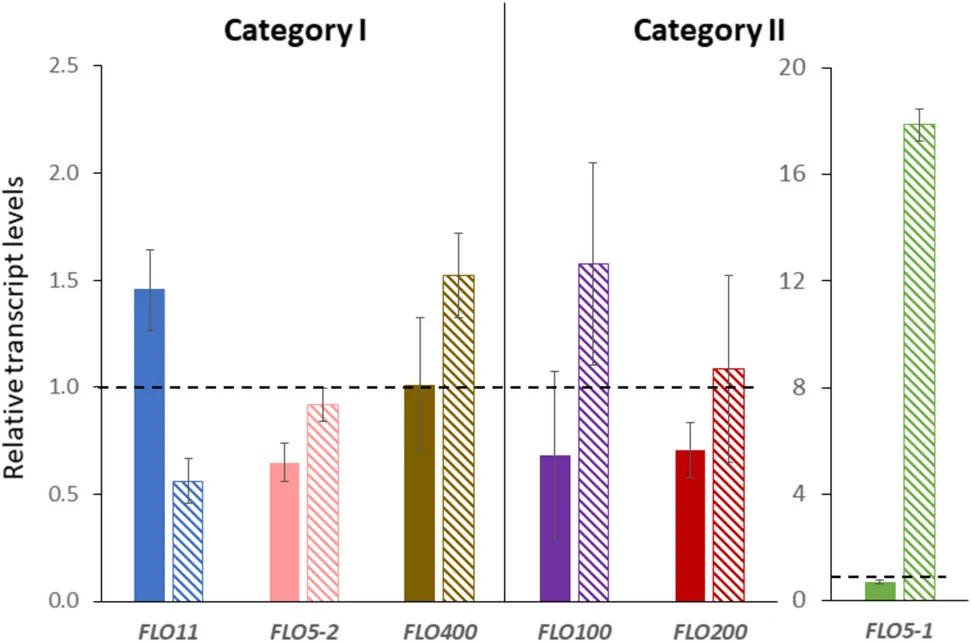
Involvement of Nrg1 in the differential regulation of *FLO *genes at pH 4.0. qRT-PCR data showing transcript levels of the six pH-sensitive *FLO* genes in the *K. phaffii NRG1*-OE (filled bars) and *nrg1Δ* (striped bars) strains, relative to the expression in the wild-type strain at pH 4.0. Please note the different scale of the *y*-axis for *FLO5-1*

To elucidate if Nrg1 was involved in *FLO* gene regulation during pH-dependent flocculation, the six *FLO* genes that displayed differential expression at pH 4.0 compared to pH 6.4 in the *K. phaffii* CBS7435 wild-type (categories I and II of Fig. 3) were now investigated in the *NRG1*-OE strain and the *nrg1Δ* strain. Transcript levels were analyzed by qRT-PCR and compared to the wild-type strain at pH 4.0 (Fig. 5).

Overexpression of *NRG1* represses the expression of all three genes belonging to category II (downregulated at pH 4.0 in the wild-type in Fig. 3), as well as *FLO5-2*. All three genes of category II also show higher expression levels in *nrg1Δ*, indicating that they are most probably direct targets repressed by Nrg1 in *K. phaffii*. *FLO5-1* is most strongly impacted by *NRG1* overexpression or deletion, respectively. *FLO400* is not affected by *NRG1* overexpression but shows elevated expression levels in *nrg1Δ.* Surprisingly, for *FLO11*, the presence of Nrg1 seems to have an effect of activation and not repression, since there was higher expression of the gene in *NRG1*-OE and lower expression in *nrg1Δ* compared to the wild-type. In contrast, *FLO5-1* is strongly repressed upon overexpression of *NRG1*, while in *nrg1Δ*, it is strongly upregulated by more than 15-fold compared to the wild-type strain.

### *flo5-1*Δ shows enhanced flocculation, while *flo11*Δ does not show a significant effect on flocculation

**Fig. 6:**
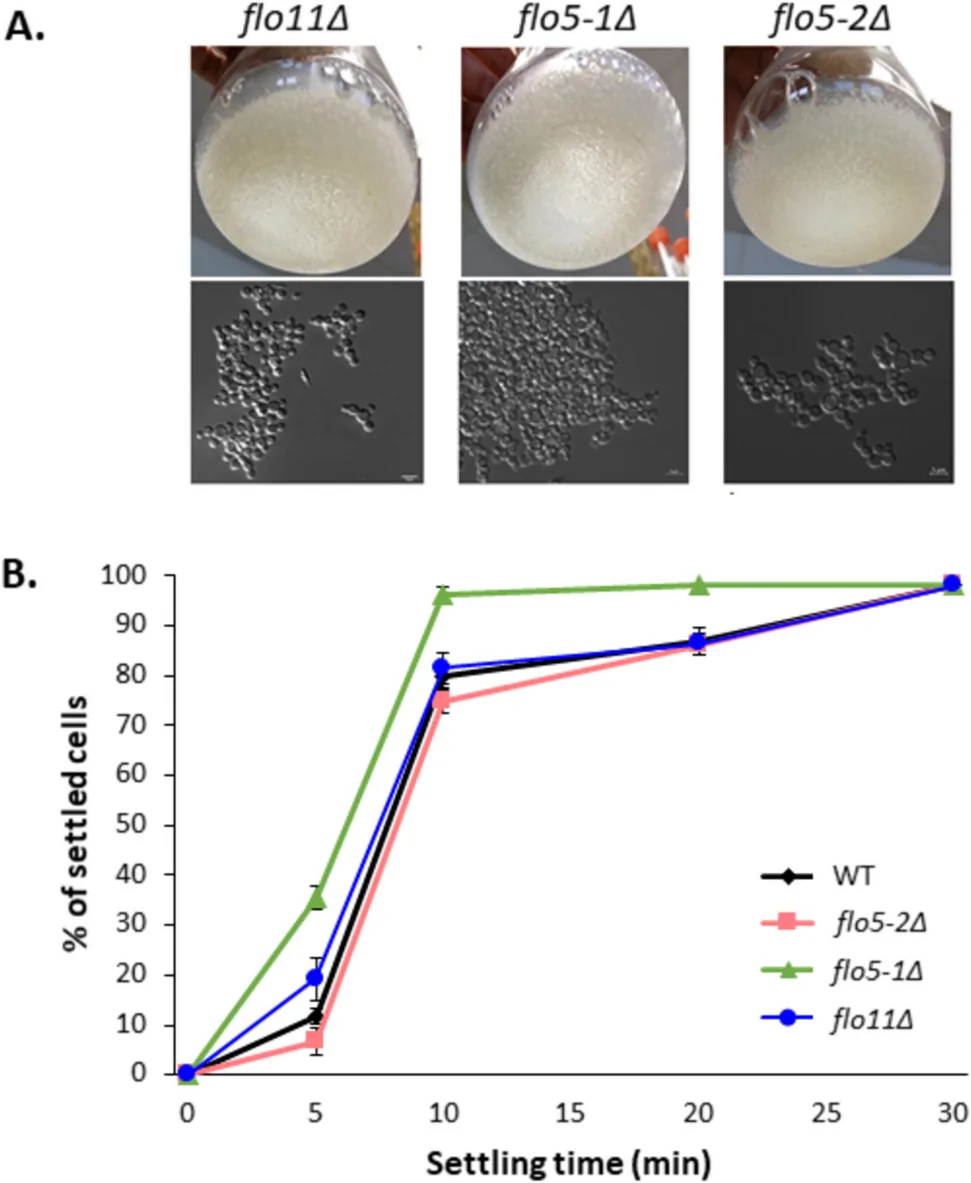
**A** Flocculation in wild-type (WT), *flo5-1*Δ, *flo5-2*Δ, and *flo11*Δ cells at pH 4.0. The top panel shows the flocculation as observed after cultivation in a shake-flask, and the bottom panel shows microscopic images from samples taken from the same flasks. **B** Sedimentation assay comparing the rate of sedimentation at pH 4.0

Based on the transcript data, the impact of deleting *FLO11* or *FLO5-1* on flocculation was analyzed. As can be seen in Fig. 6, the *flo5-1Δ* strain showed faster sedimentation and enhanced flocculation at pH 4.0. This corresponds to the qRT-PCR data since *FLO5-1* belongs to category II, that is, it shows lower expression at pH 4.0. So reduced levels of *FLO5-1* can be associated with conditions where a higher level of flocculation is observed. However, the gene also seems to be repressed by Nrg1 since it is expressed at slightly lower levels in the *NRG1*-OE strain, which showed the lowest degree of flocculation. Additionally, *K. phaffii* cells overexpressing *FLO5-1* under control of the P_*PFK300*_ promoter (approx. 15–20-fold stronger at pH 6 in glucose excess conditions based on transcriptomic data; Prielhofer et al. 2015) were generated. No difference in flocculation compared to the wild-type was observed for the *FLO5-1* overexpressing cells at pH 6.4 and pH4.0 (Supplementary Fig. S2).

Deletion of *FLO11* does not seem to have any impact on acidic pH-triggered flocculation, since the *flo11Δ* strain showed no difference in sedimentation rates compared to the wild-type. There was also no impact seen in *flo5-2Δ*, which was also included in the assays.

## Discussion

Yeast flocculation is a reversible, asexual process in which cells adhere to form flocs, and this phenomenon is mediated by cell-wall components, namely lectin-like proteins or flocculins (Willaert 2018). So far, not much is known about the phenomenon of flocculation in *K. phaffii*. We have observed that *K. phaffii* tends to flocculate at acidic pH, in particular around pH 4.0. We could show that this flocculation is reversible, and the flocs disperse upon raising or lowering the pH of the culture. Furthermore, flocculation is accelerated in the presence of high Ca^2+^ concentrations, indicating the involvement of lectin-like flocculins. Thus, this pH-dependent flocculation phenotype provided an opportunity to study the possible involvement of the *FLO* family members, which were considered the most likely candidates for imparting this phenotype based on their important role in flocculation in other yeast species. Additionally, the transcription factors Flo8 and Nrg1 were investigated for their role in this phenomenon.

Out of the 11 analyzed *FLO* gene members, *FLO11*, *FLO5-2*, and *FLO400* were higher expressed in response to low pH, while *FLO5-1, FLO100*, and *FLO200* showed lower expression at pH 4.0 compared to pH 6.4. Of these, all three flocculins showing lower expression as well as Flo5-2 contain an N-terminal PA14 lectin domain which was shown to be important for cell–cell interaction in *S. cerevisiae* (Teunissen and Steensma 1995; Caro et al. 1997; Guo et al. 2000; Essen et al. 2020). In contrast to *S. cerevisiae*, deletion of Flo8 is not sufficient to prevent flocculation in *K. phaffii*. This indicates that the transcriptional regulator has a more specialized or divergent function/s in response to environmentally triggered morphological changes in the evolutionary distant non-conventional yeast species, as was previously shown also for other transcriptional regulators in *K. phaffii* (Ruth et al. 2014; Ata et al. 2018).

Among the two investigated transcription factors, Nrg1 was revealed to be crucial for pH-dependent flocculation in *K. phaffii*. *NRG1* expression levels were lower in cells cultivated at pH 4.0 compared to pH 6. The flocculation phenotype seems to be under negative control of Nrg1, as *NRG1* overexpression significantly reduces floc formation at acidic pH. As opposed to the *NRG1-*OE, the *nrg1Δ* strain shows no impact on flocculation compared to the wild-type strain. Nrg1 acts as a global repressor and in *S. cerevisiae* is known to act by direct binding to a specific promoter region. Thus, it is likely that its overexpression magnifies its effect of repression on flocculation-triggering genes, whereas its deletion does not impact the induction of these genes. This could explain why *nrg1Δ* flocculates to a similar degree as the wild-type strain.

Further qPCR analysis of the *NRG1-OE* and *nrg1Δ* strains showed that, among the six identified pH-responsive *FLO* genes, *FLO5-1* and *FLO11* are most strongly affected by the overexpression or absence of the transcription factor. Strikingly, deletion of *FLO5-1* resulted in increased flocculation, which corresponds to the lower expression level of *FLO5-1* at low pH but contradicts the finding that *FLO5-1* is repressed in the *NRG1* overexpression strain. In contrast, no impact on flocculation was observed in cells overexpressing *FLO5-1*. Deletion of *FLO11* does not seem to have any major effect on flocculation as this strain sediments at the same rate as the wild-type strain, even though qRT-PCR showed that *FLO11* expression is activated at pH 4.0. It is possible that this activation is an effect unrelated to flocculation and *FLO11* is solely involved in pseudohyphae formation in *K. phaffii*. Since *FLO11* expression levels are generally quite low, it is possible that the twofold activation that we see at pH 4.0 compared to pH 6.4 is not enough to confer any phenotypic changes, namely elongation of cells. A strong dependence of colony morphology on *FLO11* expression levels has previously been reported in *S. cerevisiae* (Van Mulders et al. 2009).

While Nrg1 clearly has been implicated in the regulation of morphological differentiations as a repressor of invasive growth, biofilm formation, and filamentation in other yeast species, we have not found any report of Nrg1 regulating flocculation in the literature so far. In *S. cerevisiae*, the repressor Nrg1 and its paralog Nrg2 (which is absent in *K. phaffii*) were reported to function as negative regulators of haploid invasive growth and diploid pseudohyphae formation by repressing *FLO11* expression (Kuchin et al. 2002). Similarly, *Candida albicans* Nrg1 was implicated as a negative regulator of filamentous growth (Braun et al. 2001; Murad et al. 2001). Deletion of Nrg1 was implicated in increased filamentation or invasive growth in these species, and an induction of *FLO11* in *S. cerevisiae*. In contrast, in the fission yeast *Schizosaccharomyces japonicus* or the basidomycete *Ustilago maydis*, Nrg1 has been suggested as an activator of yeast-to-hyphae transition (Gómez-Gil et al. 2019; Sanchez-Arreguin et al. 2021), pointing to the evolutionary divergence of this transcription factor.

The *S. cerevisiae* Nrg1/2 were reported to interact with the kinase Snf1, which is involved in regulating a wide range of genes, including *FLO11*, in response to glucose limitation. Snf1 was reported to antagonize repression by Nrg1/2 (Cullen and Sprague 2000; Kuchin et al. 2002). A similar indirect effect of Nrg1 on *FLO11* is also assumed to occur in *K. phaffii*, as we saw repression of *FLO11* expression in *nrg1Δ* in *K. phaffii*, and even a slight induction of *K. phaffii FLO11* in the *NRG1* overexpressing strain, indicating that *FLO11* is not repressed by Nrg1 in the analyzed conditions in *K. phaffii*. So far, any attempts to generate a *K. phaffii* strain deleted for the homolog of Snf1 kinase failed (Shen et al. 2016; Barbay et al. 2021) which might be correlated to the strictly respiratory growth of *K. phaffii*. Furthermore, deletion of Flo11 does not influence the flocculation behavior.

Deletion of *flo5-1Δ* seems to increase flocculation and give rise to faster settling cells. This is also a peculiar behavior since this means that a presumed flocculin is participating in the inhibition of flocculation. This observation fits with the qRT-PCR data, since we observed a repression of *FLO5-1* at pH 4.0. Previously, it was speculated that Flo5-1 might be acting as an upstream signal for *FLO11* expression and that an initial expression of Flo5-1 is necessary for the expression of *FLO11* upon experiencing the trigger, as *flo5-1Δ* cannot express *FLO11* or transition to the pseudohyphal phenotype in response to slow growth in glucose-limited chemostats (De et al. 2020). Also, in this study, expression patterns of *FLO11* and *FLO5-1* seem to be counter-regulated. While the expression pattern of *FLO5-1* in the *NRG1*-OE strain seems contradictory to the observations in the knock-out strain in the first place, we assume that also during pH-dependent flocculation, Flo5-1 represents a sensing protein or a flocculation antagonist whose absence leads to flocculation and that its expression is repressed by Nrg1 at acidic pH. Since both pseudohyphal transition and flocculation occur in response to environmental stress, it could be speculated that in the absence of a trigger for pseudohyphal transition, a higher number of cells switch to a flocculent phenotype.

As we previously observed for pseudohyphal transition in *K. phaffii*, also in the case of acidic pH-triggered flocculation, the regulation mechanism seems to be quite complex, making the identification of the responsible *FLO* genes not straightforward. Also, there seems to be a cross-talk between multiple *FLO* genes, which still remains unclear. Previously, we have observed that in *K. phaffii*, several of the *FLO* genes were being expressed even under standard growth conditions, making it possible that in the absence of one gene, the expression of another *FLO* gene is able to confer a phenotype. In the future, further investigation should be carried out to study the function of Nrg1 in *K. phaffii* and its effect on *FLO* genes under different conditions. The *NRG1*-OE strain is a promising candidate in this respect and presents the opportunity for gaining novel insights as to how *K. phaffii* forms multicellular structures in response to environmental triggers.

## Supplementary Information

Below is the link to the electronic supplementary material.

ESM 1 (PDF 310 KB)

## Data Availability

All data supporting the findings of this study are available within the paper and its Supplementary Information.
