# Supplementary material for: Flo5-1 and Nrg1 are involved in reversible pH-dependent flocculation in Komagataella phaffii
Source: Appl Microbiol Biotechnol. 2025 Aug 4;109:178. doi: 10.1007/s00253-025-13562-7 (PMC12321659; doi:10.1007/s00253-025-13562-7)
Supplement: Supplementary file 1 [file 253_2025_13562_MOESM1_ESM.pdf]

Applied Microbiology and Biotechnology

Supplementary Information to

Flo5-1 and Nrg1 are involved in reversible pH-dependent flocculation in *Komagataella phaffii*

Sonakshi De [1,2,3], Gerhard Stadlmayr [2$], Corinna Rebnegger[1,2], Diethard Mattanovich [1,2], Brigitte Gasser [1,2,3*]

[1] Austrian Centre of Industrial Biotechnology, Vienna, Austria

[2] Department of Biotechnology, Institute of Microbiology and Microbial Biotechnology, BOKU University, Vienna, Austria

[3] Christian Doppler Laboratory for growth-decoupled protein production in yeast, Department of Biotechnology, BOKU University, Vienna, Austria

Present address:

$ Ablevia biotech GmbH, Maria Jakobi Gasse 1, 1030 Wien, Austria

Corresponding author:

Brigitte Gasser

BOKU University, Department of Biotechnology, Institute of Microbiology and Microbial Biotechnology,

Muthgasse 18, 1190 Vienna, Austria

brigitte.gasser@boku.ac.at

Supplementary Table S1: List of *K. phaffii* strains used or generated in this study

Strain	Genotype	Resistance	
**CBS7435**	Wild-type		
**CBS7435 *flo8Δ***	*flo8Δ*	none	Rebnegger et al. 2016;<br>De et al. 2020
**CBS7435 *flo11Δ***	*flo11Δ::loxP-natMX-loxP*	Nourseothricin	De et al. 2020
**CBS7435 *flo400Δ***	*flo400Δ::loxP-kanMX-loxP*	Geneticin	De et al. 2020
**CBS7435 *flo5-1Δ***	*flo5-1Δ::loxP-kanMX-loxP*	Geneticin	De et al. 2020
**CBS7435 *NRG1*-OE**	CBS7435 P_{GAP}-*NRG1*	Nourseothricin	This study
**CBS7435 *nrg1Δ***	*nrg1Δ::loxP-natMX-loxP*	Nourseothricin	This study
**CBS7435 *flo5-2Δ***	*flo5-2Δ::loxP-kanMX-loxP*	Geneticin	This study
**CBS7435 *FLO5-1*-OE**	CBS7435 P_{PFK300}-*FLO5-1*	Nourseothricin	This study

Supplementary Table S2: Primers for Golden Gate Assembly of deletion and overexpression cassettes. The restriction sites for Golden Gate Cloning are underlined.

flo5-2Δ	5' homologous region FS AB	GGTCTCCGATCTACGTATCCTCCATCCTGATCGC<br>GGTCTCCCCGGTTAATATGAGCGACAGGTGGAGT
	3' homologous region FS CD	GGTCTCTAATTCTGAACGTACATTCATTTCAGGTG<br>GGTCTCCAGCTAACTTGAAGGACTGAAAGCACCCG
nrg1Δ	5' homologous region FS AB	GGTCTCCGATCGTTGCCGACAGATTTGTTGTTCAATG<br>GGTCTCCCCGGGACAAAGTAGGATTCAACAGCTATTCTG
	3' homologous region FS CD	GGTCTCTAATTGTATTTATTTACGGATTGGACCCAAGCGAG<br>GAGTAAGTAGTATG<br>GGTCTCCAGCTCAAATTGAGTACCTCTATCCAATACTAAGTT<br>GAATTCGACATATCTTC
NRG1-OE	Promoter P_{GAP}	GGTCTCTGATCAGGATCCTTTTTTGTAG<br>GGTCTCTCCGGGTGTTTTGATAG
	Gene *NRG1*	GGTCTCTCCGGATGAGTTTTCAAGTCTACAACATGGTGTC<br>GGTCTCTAATTCTATTTACTTCTCAATCTAGATCTCTTTCGTTTCGAC
	Terminator RPS25ATT	GGTCTCTAATTTTAGTGTACATCTG<br>GGTCTCTAGCTGTCAAATATTCAGATG
Marker genes with *lox* sites	FS BC	GATAGGTCTCCCCGGGTACGCTGCAGGTCGACAAC<br>GATAGGTCTCCAATTAGTGGATCTGATATCACCTA
	FS 23	GATAGAAGACTCCATGGTACGCTGCAGGTCGACAAC<br>GATAGAAGACTCAAGCAGTGGATCTGATATCACCTA

Supplementary Table S3: Primers for amplification of split marker cassettes and verification of positive deletion/overexpression in the *K. phaffii* genome

flo5-2Δ	FLO5-2_KO_ampl_fwd	TACGTATCCTCCATCCTGATCGC
	KanMX_rev	CAGGAACACTGCCAGCGCATCAAC
	KanMX_fwd	GATGTTACAGATGAGATGGTCAGAC
	FLO5-2_KO_ampl_rev	AACTTGAAGGACTGAAAGCACCCG
	FLO5-2_KO_ctr_fwd	TGGCGTTTTACGTATCCTCCATC
	FLO5-2_KO_ctr_rev	AGTTTAGTGGACAAAGAGTACTGAATG
nrg1Δ	NRG1_KO_ampl_fwd	GTTGCCGACAGATTTGTTGTTCAATG
	KanMX_rev	TGCGTTGACGTTGGTGAC
	KanMX_fwd	GTCCTTCACCACCGACACC
	NRG1_KO_ampl_rev	CAAATTGAGTACCTCTATCCAATACTAAGTTG
	NRG1_KO_ctr_fwd	GAATCACTAACCAGGGTCAATGAATTGAG
	NRG1_KO_ctr_rev	CAGCTGTTCTTGGCAACGACATCAAAATAC
NRG1-OE	P_{GAP}_fwd	CGTGTACCCGACCTAGCAGCCCAG
	RPS25Att_rev	GGAGGATCTTGAAATGTTAGGGTC
FLO5-1-OE		

Supplementary Table S4: qRT-PCR primers for transcript analysis.

FLO11	AGTCCACACACCATTGACAACTGC
	ACACCAGTAACTATTGTAGCAACTGAGC
FLO5-1	CAGGCAAGTGATGACAATTCAATACAGGAC
	CGTTTGGTATCCCATAAAGTGTTGTGGTG
FLO5-2	GCTATTTCAAAGCTGCTGTTTCAGGAGAC
	AGCGAATAGTCTGTTGGTGCTTGATCC
FLO400	GGAGCTGAAGGTTCGGTAGAAT
	GTTCTGCACCCTCACAAATGT
FLO100	TCACGACCGGTGGTACGACTA
	ATTTGGCACAACGTGGCTAGGT
FLO200	GTCTGGGACAAGTGACAACA
	ATGGTTGCATTCACGTACGA
BSC1	TGAGGAATCCACAGAGGAATCCACAT
	TGGATTCCTCAGCATCCTCAGTAGA
ACT1	CCTGAGGCTTTGTTCCACCCATCT
	GGAACATAGTAGTACCACCGGACATAACGA

Gene ID	Protein name
**PP7435_Chr2-0267**	Flo11
**PP7435_Chr1-1389**	Flo5-1
**PP7435_Chr3-1228**	Flo5-2
**PP7435_Chr1-1587**	Flo100
**PP7435_Chr3-1226**	Flo200
**PP7435_Chr4-1020**	Flo300
**PP7435_Chr4-0865**	Flo400
**PP7435_Chr4-0629**	PP7435_Chr4-0629
**PP7435_Chr4-1013**	PP7435_Chr4-1013
**PP7435_Chr1-1549**	Bsc1

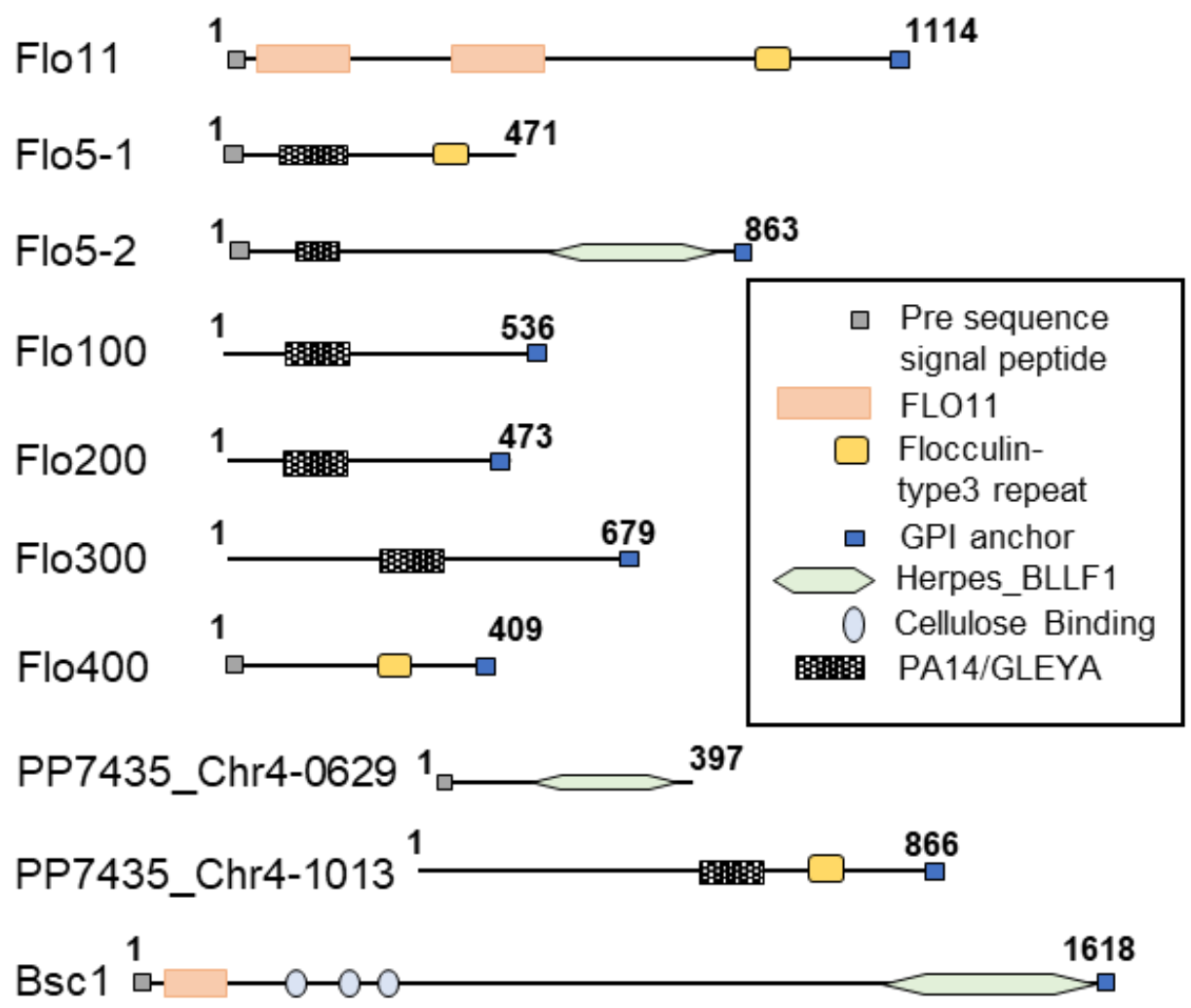

Supplementary Fig. S1: The *FLO* gene family of *K. phaffii* (adapted from De et al. 2020)

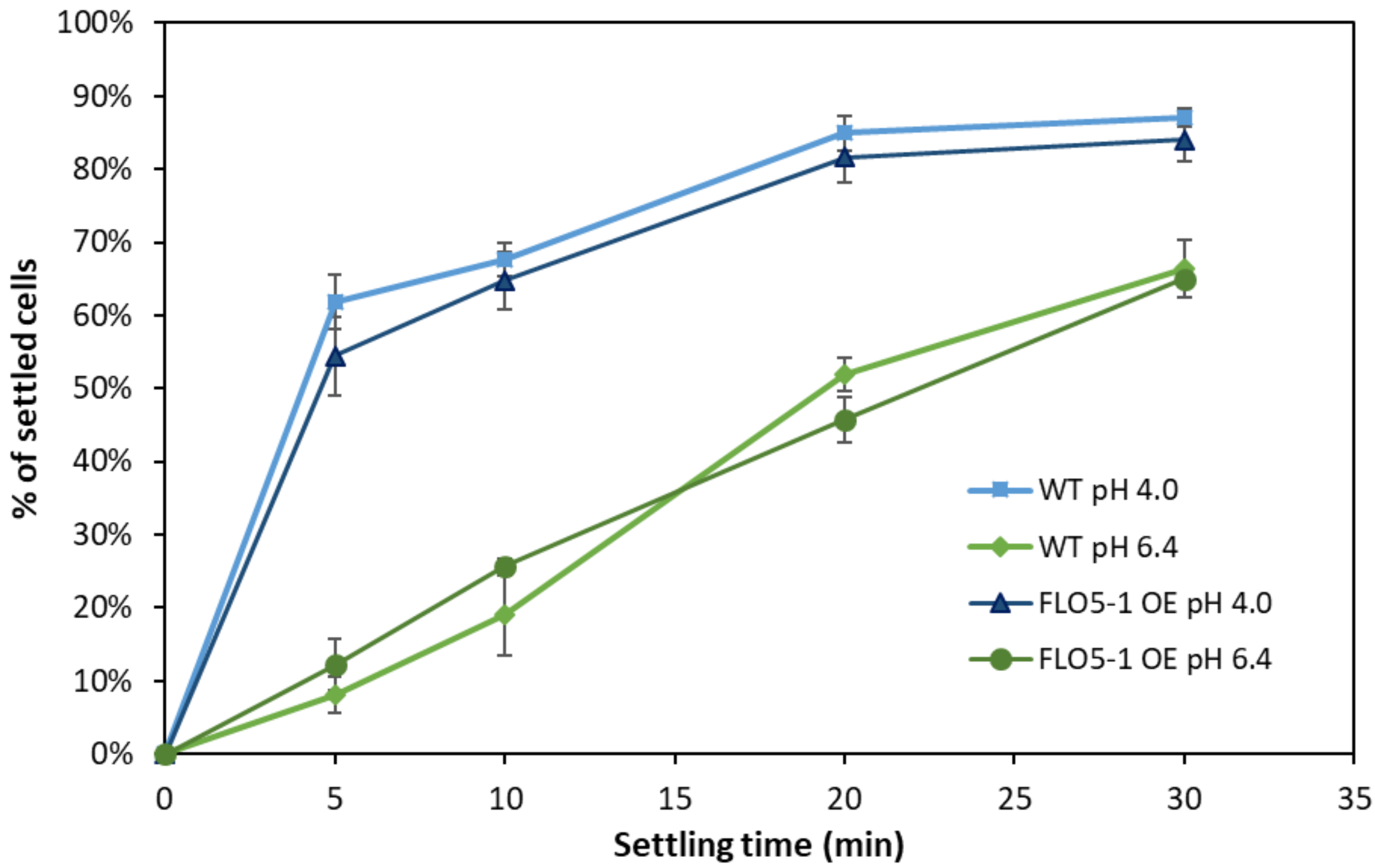

Supplementary Fig. S2: Impact of *FLO5-1* overexpression on pH-dependent flocculation in *K. phaffii*. Sedimentation assay comparing the rate of sedimentation of wild-type *K. phaffii* CBS7435 without (WT) or with overexpression of *FLO5-1* under the control of the P_{PFK300} promoter (FLO5-1 OE) at different pH. Error bars show standard deviation of three individual experiments.

References:

De S, Rebnegger C, Moser J, Tatto N, Graf AB, Mattanovich D, Gasser B (2020) Pseudohyphal differentiation in *Komagataella phaffii*: investigating the *FLO* gene family. FEMS Yeast Res 20:foaa044

Rebnegger C, Vos T, Graf AB, Valli M, Pronk JT, Daran-Lapujade P, Mattanovich D (2016) *Pichia pastoris* exhibits high viability and a low maintenance energy requirement at near-zero specific growth rates. Appl Environ Microbiol 82:4570-4583
